# Human Milk Banking In Nepal: An Initiative To Strengthen Newborn Nutrition

**DOI:** 10.31729/jnma.v63i290.9198

**Published:** 2025-09-01

**Authors:** Smriti Poudel, Kalpana Upadhaya Subedi, Shailendra Bir Karmacharya, Prajwal Paudel, Rina Shrestha, Punam Maharjan, Laxmiswori Prajapati

**Affiliations:** 1 Paropakar Maternity and Women’s Hospital, Thapathali, Kathmandu, Nepal

**Keywords:** *breastfeeding*, *milk banking*, *human milk*

## Abstract

To address the gap in suboptimal breastfeeding practices and limited access to human milk and its challenges to neonatal health outcomes, the country’s first Human Milk Bank, “Amrit Kosh,” was established at Paropakar Maternity and Women’s Hospital (PMWH) in August 2022. This study presents the experience of its operation, highlighting development, induction, challenges, and future directions for the program. Data were extracted from Human Milk Bank procedure records. Standard quality assurance protocols were followed throughout milk handling and processing, adhering to national guidelines. Of 2859 willing donors, 2820 mothers met eligibility criteria, contributing 1760.35 liters of breast milk. All batches of pasteurized donor human milk (PDHM) passed bacteriological screening, ensuring zero contamination, which was dispensed to 3511 neonates.

The successful establishment and safe operation of Nepal’s first Human Milk Bank demonstrate the feasibility and impact in a resource-limited setting. Future priorities include securing sustainable funding, expanding community engagement, and scaling the model to other facilities. “Amrit Kosh” has provided critical nutrition while enhancing breastfeeding awareness.

## INTRODUCTION

Improving newborn care globally has highlighted the need for sustainable, cost-effective models like Human Milk Banking (HMB).^1^ Breast milk, the gold standard for infant nutrition and immunity, significantly reduces child mortality^2^, yet only 38% of infants are exclusively breastfed worldwide.^3,4^ In Nepal, rates remain suboptimal, with just 56% exclusively breastfed for six months.^5^ Vulnerable newborns especially preterm, low birth weight, or orphaned benefit from donor human milk (DHM) when their mother’s own milk is unavailable. “Amrit Kosh,” Nepal’s first HMB at Paropakar Maternity and Women’s Hospital, addresses both milk donation and lactation support, ensuring equitable access to lifesaving breast milk.

### Need for the Human Milk Bank at Paropakar Maternity and Women’s Hospital.

Paropakar Maternity and Women’s Hospital (PMWH) is a tertiary level maternity hospital and referral center in Nepal. More than 20000 deliveries are conducted annually, including 30-40% of caesarean section deliveries. Total Low Birth Weight (LBW) deliveries is 11.14% and preterm deliveries is 3.6%. More than 200 newborns are admitted to Special New born Care Unit (SNCU) and Neonatal Intensive Care Unit (NICU) monthly, new-boms with LBW, preterm and premature new-boms and neonatal sepsis causing most of the burden. About 35-40% of the new-borns admitted to NICU and SNCU are in need of Donor Human Milk (DHM) during their stay.^6^ Prematurity or sickness, mother’s illness, lactation failure in mothers, or circumstances like abandonment and death of the mother may deprive newborn babies of mother’s milk.

### Development and Establishment of the first Human Milk Bank in Nepal.

Paropakar Maternity and Women’s Hospital (PMWH), a designated Baby Friendly Hospital, has long championed optimal infant feeding practices. With all faculty and nursing staff trained in lactation management and several serving as national Master Trainers, PMWH plays a leading role in advancing breastfeeding, Kangaroo Mother Care (KMC), and newborn care protocols.

Despite these ongoing efforts, challenges persist in ensuring exclusive breastfeeding, particularly for preterm and low birth weight infants. In critical care settings such as NICU and SNCU, formula feeding was often the only alternative when a mother’s own milk was unavailable due to caesarean recovery or lactation failure. The lack of facilities for milk expression and access to Pasteurized Donor Human Milk (PDHM) underscored the urgent need for a Human Milk Bank (HMB).

In response, and alignment with updates to Nepal’s Breast Milk Substitutes Act, a landmark decision was made during the Breastfeeding Protection and Promotion Sub-Committee meeting on March 16, 2021, to establish the country’s first HMB at PMWH. This initiative was supported by the Ministry of Health and Population (MoHP), the Department of Health Services (DoH), and the Family Welfare Division (FWD), with technical support from the UNICEF Nepal, the EU, and USAID.

On August 2022, “Amrit Kosh” Nepal’s first Human Milk Bank was officially inaugurated at PMWH. It operates as a nonprofit service, collecting surplus breast milk from screened, healthy donors and supplying PDHM to premature and ill newborns. A multidisciplinary hospital-based team, including experts in neonatology, microbiology, nursing, and lactation, oversees its operations.

Amrit Kosh not only addresses a critical gap in neonatal nutrition but also reinforces national priorities around newborn survival and breastfeeding support. Its establishment marks a transformative step in ensuring that even the most vulnerable infants receive the best possible start in life.

## INDUCTION AND AWARENESS

To build technical competency, MoHP, DOH/FWD, UNICEF Nepal and PATH had a two day learning exchange with the HMB team of PMWH. All HMB processes were shared, including all aspects of quality control regarding collection, screening, storage, and pasteurization, as well as the prioritization and appropriate use of PDHM in the neonate unit, donor recruitment, and the provision of lactation support (including early initiation of breastfeeding or milk expression and frequent expression for building maternal milk supply). Additional operational aspects, including staffing structure, costing and sustainability were also reviewed.

Following two days training on Human milk banking, an orientation session to all the hospital staffs regarding the HMB and breastfeeding was held in 10 batches and included doctors, nursing staffs, administration and support staffs. Lactation support was strengthened for all mothers at the hospital by training hospital staff for improved breastfeeding, milk expression, and milk storage practices. Additionally, the availability of the HMB services and access to the HMB facility created additional touch points for lactation support. Overall strengthening of lactation support included key messages for early and frequent breastfeeding or milk expression, promoting the mother to visit and be present as often as possible, the use of mother’s own milk when possible.

## OPERATIONAL OVERVIEW

**Figure 2:**
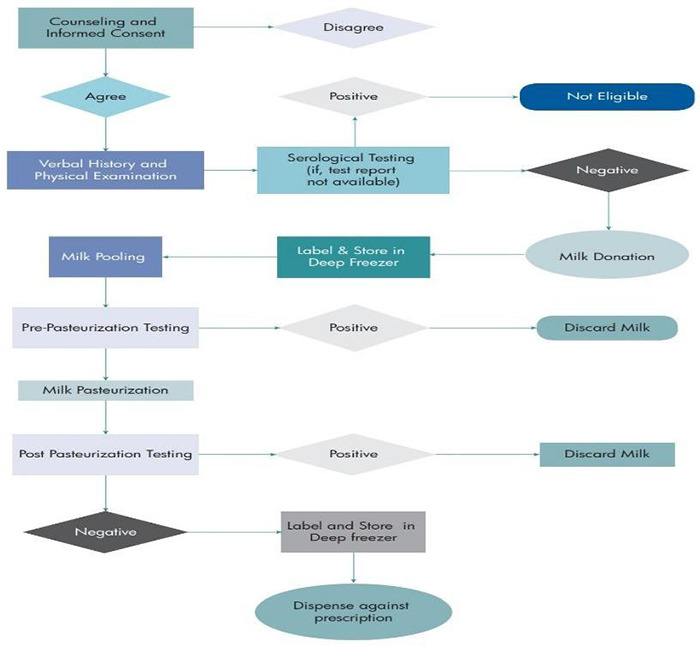
Flow of process practices in Human Milk Bank at Paropakar Maternity and Women’s Hospital (PMWH)

Human Milk Bank performs numerous operational processes to provide safe, high-quality donor milk. Figure 2 is an operational flow chart showing stepwise practices performed. These process steps, or standards of practice, start with the recruitment of donors, continue with the handling and processing of donor human milk, and finish with the allocation of the donor milk to the recipient. The milk bank has adopted standards of practice laid down by the guideline on Comprehensive Lactation Management Centres (CLMC)^7^ by the Family Welfare Division, Department of Health.

The data from July 2022 to July 2025 was extracted from procedure record book of the HMB, donor recruitment record files and Milk requisition record register of recipients of the milk bank. The total number of mothers who were willing to donate were 2859, out of which only 2820 were eligible after passing the screening procedure of HMB. The primary reason for the disqualification was serology reactive cases followed by breast abscess in mother. Around 1760 liters of milk was collected, 1726 liters was pasteurized and 1675 liters of the pasteurized milk was dispensed to 3511 new-borns in the NICU and SNCU of PMWH and needful new-borns from other hospitals in Kathmandu.

Standard quality assurance protocols laid down by the National Guideline on Comprehensive Lactation Management Centres were followed throughout the process and reviewed monthly by the Hazard Analysis and Critical Control Point Committee (HACCP). Both pre-pasteurization and post-pasteurization culture test was done on each batch of the milk from the beginning of the operation of HMB at the hospital’s central laboratory. The passing rate of the bacteriological test was 100 percent. Donor human milk is not accepted from the community as of now and utmost precautions for the hygiene and sterile conditions of the settings is taken care of.

## CHALLENGES FACED DURING IMPLEMENTATION AND OPERATION

Human Milk Banks (HMBs) have existed for over a century, with the first established in Austria in 1909. While many developed countries and neighbouring India adopted this concept early, India starting 33 years ago and Nepal only initiated serious policy discussions in 2020 A.D., making it a late entrant in this important healthcare innovation.

Challenges faced during the first year of its functioning included; financial and time costs of doing the intervention for the first time in a country, without access to ongoing technical monitoring and support structures or examples in the proximal area. Ongoing costs for HMB operations continue to be challenging. Securing sustainable funding and resources to maintain operations is a huge challenge for this site. Some other challenges are capacity building to develop a well-trained pool of workforce as a pioneer in the field and retention of the workforce in the scenario of health professionals migrating for opportunities.

**Table 1:** Number of Donors, Volume of milk collected, Pasteurized and Dispensed and Number of Recipients at Paropakar Maternity and Women’s Hospital, Human Milk Bank.

Variables	Year (A.D)			Total
	2022/23	2023/24	2024/25	
Total Number of qualified Donors	785	1161	874	2820
Total number of Recipients	873	1673	965	3511
Total amount of milk collected in donation	451.59 litres	777.48 liters	531.265 litres	1760.35 liters
Total amount of milk pasteurized	448.84 liters	682.44 liters	595.10 liters	1726.43 liters
Total amount of milk dispensed	440.69 liters	658.89 liters	575.45 liters	1675.03 liters

**Table 2:** Phased activities for the establishment of Comprehensive Lactation Management Centres at Paropakar Maternity and Women’s Hospital.

Phase One: Establishing a Foundation	Phase Two: Operationalize and Stabilize	Phase Three: Research and Evaluation
Committee formation	Orientation sessions to the staffs	Conduct rigorous evaluation
Preliminary assessment and survey	Breast feeding promotion activities	Disseminate findings to ensure sustainable expansion
Recruitment	Establishment of quality control system	Revise system based on evaluation
Capacity building and learning exchanges	Implementation of integrated human milk bank program along with lactation management program	
Provision of intensive mentorship and skill based training for HMB staffs		

Adequate donor recruitment and balancing the demand and supply with the existing resources are ongoing challenges. Besides, educating healthcare professionals, mothers and other general public about the benefits of donor milk and the need for donations can be an ongoing challenge.

Despite, being a multicultural country with prevalence of myths and taboos around breastfeeding and new-born care, no sociocultural hindrances and barriers were encountered throughout this period.

(Adopted from A Resource Toolkit for Establishing and Integrating Human Milk Bank Programs: A Global Implementation Framework. Version 2.0.)^8^

## FUTURE DIRECTION

### Community Awareness and Donor Recruitment

Collaborate with local community leaders, healthcare workers, and media outlets to raise awareness about the milk bank's services and benefits. Robust donor recruitment from the community

### Collaboration with Healthcare Institutions

Partner with hospitals, clinics, and midwifery services to create a seamless referral and distribution network. Offer training programs for healthcare professionals on the benefits and protocols for using PDHM.

### Research and Development

Conduct research to study the impact of donor milk on neonatal health outcomes in Nepal, particularly in preterm and low-birth-weight infants. Explore innovations in milk preservation, pasteurization techniques, and storage methods to optimize efficiency. Generation of evidences for future practices in Human Milk Banking through research activities.

### Regulatory Framework and Accreditation

Seek international accreditation and adherence to global standards

### Sustainability and Funding

Develop a sustainable funding model through partnerships with NGOs, government health programs, and international health organizations.

### Integration with Maternal and Child Health Programs

Align the milk bank’s activities with national maternal and child health initiatives, such as breastfeeding promotion and nutrition programs. Use the milk bank as a platform to provide breastfeeding education and support for mothers.

## CONCLUSION

Despite facing obstacles such as logistical issues, expertise in the field and the need for extensive community education, the human milk bank has proven to be an essential service in addressing neonatal malnutrition and providing vulnerable infants with the best start in life. This experience demonstrates the feasibility of establishing and sustaining human milk banks in resource-limited settings like Nepal, with the potential to save new-borns lives. Moving forward, it is crucial to expand public awareness, strengthen infrastructure, and foster local partnerships to ensure the long-term sustainability and growth of milk banking services. Continued research and adaptation to local contexts will be vital for scaling these efforts to reach more communities in need, ultimately improving infant health outcomes across the country.
